# Primary Phase Field of the *Pb*-Doped 2223 High-T_c_ Superconductor in the *(Bi, Pb)-Sr-Ca-Cu-O* System

**DOI:** 10.6028/jres.104.020

**Published:** 1999-06-01

**Authors:** W. Wong-Ng, L. P. Cook, A. Kearsley, W. Greenwood

**Affiliations:** National Institute of Standards and Technology, Gaithersburg, MD 20899-0001; University of Maryland, College Park, MD 20742

**Keywords:** high-*T*_c_ superconductor, Pb-doped 2223, BSCCO, phase equilibria, primary phase field

## Abstract

Both liquidus and subsolidus phase equilibrium data are of central importance for applications of high temperature superconductors in the (Bi, Pb)-Sr-Ca-Cu-O system, including material synthesis, melt processing and single crystal growth. The subsolidus equilibria of the 110 K high-*T*_c_ Pb-doped 2223 ([Bi, Pb], Sr, Ca, Cu) phase and the location of the primary phase field (crystallization field) have been determined in this study. For the quantitative determination of liquidus data, a wicking technique was developed to capture the melt for quantitative microchemical analysis. A total of 29 five-phase volumes that include the 2223 phase as a component was obtained. The initial melt compositions of these volumes range from a mole fraction of 7.3 % to 28.0 % for Bi, 11.3 % to 27.8 % for Sr, 1.2 % to 19.4 % for Pb, 9.8 % to 30.8 % for Ca, and 17.1 % to 47.0 % for Cu. Based on these data, the crystallization field for the 2223 phase was constructed using the convex hull technique. A section of this “volume” was obtained by holding two components of the composition at the median value, allowing projection on the other three axes to show the extent of the field.

## 1. Introduction

The rapid pace of applied research on the high-*T*_c_ superconductors has continued since their initial discovery in the Bi-Sr-Ca-Cu-O (BSCCO) system [1]. To date, many prototype products have been developed, and large scale applications are possible [2]. In the Pb-free BSCCO system, it is well known that it is relatively easy to prepare the 80 K 2212 (Bi:Sr:Ca:Cu) compound in nominal single-phase form. By contrast, the processing window for the Pb-free 2223 phase is narrow, and obtaining a commercially significant amount of single-phase material is difficult. Partial doping of Bi with Pb, on the other hand, can stabilize the phase formation of the Pb-doped 110 K 2223 ([Bi, Pb]:Sr:Ca:Cu) superconductors [5–10]. Therefore, industrial processing of high-*T*_c_ superconductor materials has focused primarily on advancing the commercial potential of the Pb-free 2212 and the Pb-doped 2223 superconductors for wire and tape applications. For simplicity, in this paper, we will refer to the Pb-doped phase simply as 2223.

### 1.1 Powder-in-Tube Processing Technique

In recent years, various fabrication techniques of the BSCCO 2212 and 2223 superconductor wires and tapes were developed [11–14]. Among them, the powder-in-tube (PIT) technique was found to be most viable. This technique involves a multistep process of filling Ag tubing with high-*T*_c_ BSCCO powder, followed by repeated packing, cold drawing, rolling, and thermal processing. The presence of a liquid phase enhances the growth and preferred orientation of micaceous superconductor grains, and often leads to grain alignment. Significantly improved superconducting properties as a result of this melting/alignment phenomenon, and of the presence of Ag, were obtained [15–21]. The tapes produced by these methods have demonstrated a capacity to support high critical currents in high magnetic fields [3,22,23]. Therefore, the PIT technique offers a promising route to the industrial-scale fabrication of long-length, high-quality superconducting cables for electric power and high-field magnetic applications. These BSCCO superconductors show further improvements in properties with the introduction of artificial pinning centers [24, 25].

### 1.2 Phase Equilibrium Data

Phase diagrams provide fundamental processing maps, and it is therefore essential to have data on all aspects of the phase relationships in the BSCCO system, including solid-state homogeneity regions, melting equilibria, and the location of the primary phase crystallization fields of the high-*T*_c_ phases in the BSCCO system. Information on melting and on the primary phase field is an invaluable guide in the optimization of 2223 ceramics through melt processing, and it also provides a framework for the interpretation of transient and metastable liquids. To date, while melt data are relatively plentiful for the Pb-free 2212 phase [26–28], only limited data are available for the Pb-doped 2223 phase [29–33].

#### 1.2.1. Phase Equilibrium Data

Because of the importance of the 2223 phase in the high-*T*_c_ industry, a relatively extensive amount of research has been conducted. These studies included the mechanisms and kinetics of 2223 phase formation [34–48], phase formation in reaction couples [49], the location of the 2223 homogeneity region [50–56], the thermal stability of the Pb-2223 phase as a function of oxygen partial pressure [57–58], and the influence of oxygen partial pressure and reaction time on the formation of the 2223 phase [69–75]. With regard to phase diagram studies, because the Bi-Pb-Sr-Ca-Cu-O system is a five-component oxide system, a complete investigation requires extensive effort. Thus far, many studies have concentrated on small regions pertaining to the Pb-2223 phase [56, 76–86]. For example, Toledano et al. [76] treated 2223 equilibria with reference to the quasi-quaternary (Bi_1.8_Pb_0.4_)O*_x_*–CaO-SrO-CuO system. Strobel et al. [77, 78] studied the phase diagram of the Bi_1.6_Pb_0.4_Sr_2_CuO_6_-CaCuO_2_ system, which includes Bi_1.6_Pb_0.4_SrCa*_n_*_−1_Cu*_n_*O_2n+4+*z*_, at temperatures between 825 °C and 1000 °C. Osamura and Maruyama [79] investigated phase relationships in Ag/2223 tapes and found that the 2223 phase formed only between 830 °C and 870 °C. They constructed projections of the phase equilibria on isothermal quasi-ternary Bi_2_O_3_-(SrO+ CaO)-CuO sections. Kaesche et al. [56, 80] discussed isothermal sections at 850 °C and 865 °C, using CuO (mole fraction fixed at 37 %), SrO, CaO and (Bi/Pb)_2_O_3_ as components. MacManus-Driscoll and Yi [86] reported the phase equilibria near (Bi, Pb)-2223 as a function of oxygen partial pressure. In their study, the phases in equilibirum with 2223 are in general in agreement with Kaesche et al. [56, 80] and Wong-Ng et al. [87] except for the (Ca, Sr) CuO_2_ and 119 × 5 phases (In these symbols, *x* is used to represent the amount of Ca being substituted into the Sr site).

To date, many reported equilibria are expressed, as a convenient way, as projections made by combining Bi and Pb, or Ca and Sr. However, since these elements do not substitute ideally for each other, or to the same extent in all of the compounds that are in equilibrium with the 2223 phase, it is not totally accurate to represent the results in this manner.

Presently, there is still a need for data to complete the subsolidus equilibria and the primary crystallization field of the 2223 phase. The primary objective of the NIST high-*T*_c_ phase diagram project is to develop the portions of the phase diagrams that are relevant to the processing of the 2223 compounds. This paper summarizes the determination of a complete set of the five-phase subsolidus equilibrium assemblages that contain the 2223 phase, and the determination of the primary phase field of the 2223 phase. These studies were conducted under a volume fraction of 7.5 % O_2_/92.5 % Ar atmosphere.

#### 1.2.2 Previous Work at NIST

##### 1.2.2.1. Phases in Equilibrium With the Pb-2223 Phase

Bernik has studied the influence of starting composition (Bi_2+d–_*_x_*Pb*_x_*Sr_2_Ca_2_Cu_3_O*_z_*) on the formation of the “pure” 2223 phase. A single phase region for 2223 was mapped on a plot of the mole fraction of Pb versus (Bi+Pb) [88]. In samples with nominal composition of Bi_2+_*_d_*_–_*_x_*Pb*_x_*Sr_2_Ca_2_Cu_3_O*_z_* (*d* = 0), 2223 forms when *x* ≥ 0.2. The 2223 phase coexists with 2212 in the range 0.2 ≤ *x* ≤ 0.4 after annealing at 855 °C for 100 h. Some (Ca,Sr)_2_CuO_3_ was also found. When *x* > 0.4, 2223 along with (Pb, Bi)_1.4_(Sr, Ca)_3_Cu_0.77_O*_z_* were found in these samples. The 2223 phase can be formed from starting mole fraction compositions with less than 10 % Bi replaced by Pb, provided (Bi + Pb) > 2. The 2201 phase coexists with 2223 when (Bi + Pb) > 2.3 and Pb > 0.1. As a summary, the single 2223 phase region in samples with composition Bi_2+_*_d–x_*Pb*_x_*Sr_2_Ca_2_Cu_3_O*_z_* is proposed as 0.3 < *x* < 0.45, 0.05 ≤ *d* ≤ 0.35, 2.05 < (Bi, Pb) < 2.35.

In a subsequent separate study, compositions that are in equilibrium with the 2223 phase were determined [87]. From extensive x-ray diffraction results, 11 phases which include binary, ternary, and quaternary oxides were found to be in equilibrium with the 2223 phase at 810 °C to 820 °C (Table 1) [87]. In order to describe phase equilibria of various phases, it is useful to designate the complicated BSCCO formulas with simplified abbreviations, which are presented in Table 1. These designations are strictly for convenience, and are not meant to precisely convey the stoichiometric formulas. The equilibrium phases were (Ca, Sr)O, CuO, 0*x*21 ([Ca, Sr]_2_CuO_3_), 119*x*5 ([Bi,Pb]_2.2_Sr_1.8–_*_x_*Ca*_x_*CuO*_z_*), 014*x*24 (Sr_14–_*_x_*Ca*_x_*Cu_24_O_41_), 2310 (Bi_2_[Sr, Ca]_4_)O*_z_*, 2201 (Bi, Pb)_2_Sr_2–_*_x_*Ca*_x_*CuO*_z_*, 0*x*11 (Sr_1–_*_x_*Ca_x_)CuO_2_, Ca-rich and Ca-poor), 1*x*20 ([Ca, Sr]_2_PbO_4_), and 3221 ([PbBi]_3_Sr_2_Ca_2_CuO*_z_*).

The 014*x*24 phase is a solid solution in which Ca can substitute into the Sr site up to *x* = 7 [89, 90]. The 3221 phase is referred to as the Bi-doped “451” solid solution by Daesche et al. [56, 80]. It was initially discovered by Kitakuchi et al. [91] and studied in detail by Luo et al. [92]. The Ca_2_PbO_4_ (1*x*20) phase was found to form an extensive solid solution with Sr_2_PbO_4_. A complete solid solution and the Rietveld refinement studies of (Ca, Sr)_2_PbO_4_ have been reported by Kitakuchi et a. [91] and Wong-Ng et al. [93, 94]. Calcium was found to incorporate into the Raveau 11905 phase [95], to give a solid solution with the general formula of Bi_2. 2 +_ *_x_* Sr_1.8–_*_x–y_*Ca*_y_*Cu_16_*_w_*O*_z_* (119*x*5), where 0 < *x* < 0.5. Both the 119*x*5 and 2201 phases can be indexed on different monoclinic cells [96]. Because of the close proximity of these two phases, they will not be distinguished and will be referred to as 119*x*5. The 2310 solid solution has an approximate formula of Bi_2_(Sr, Ca)_4_O*_z_* and exists in high and low-temperature forms [97]. The high-temperature form, which is in equilibrium with the 119*x*5, 2212, and 2223 superconductors, is monoclinic, with space group Pc. Both the two different structure types of (Ca, Sr)CuO_2_ (0*x*11 and 0*x*11′) [98] were found to be in equilibrium with Pb-2223. The Ca-rich 0*x*11 phase is orthorhombic, whereas the 0*x*11′ phase is tetragonal.

##### 1.2.2.2 Extent of Pb-Substitution

In order to study Pb incorporation in various Bi-containing compounds, a series of samples of the 2212, 4805, 119*x*5, 2201, and 2310 phases was prepared by assuming the same Bi/Pb ratio as in the 2223 phase, namely 1.8/0.4 [87]. These samples have stoichiometry of Bi_27.82_Pb_6.18_Sr_49.5_Ca_16.5_O*_x_*(2310), Bi_1.8_Pb_0.4_Sr_1.6_Ca_0.2_ CuO*_x_* (119*x*5), Bi_1.64_Pb_0.36_Sr_2_CuO*_x_* (2201), Bi_1.64_Pb_0.36_ Sr_1.5_Ca_1.5_Cu_2_O_x_ (2212), and Bi_3.28_Pb_0.72_Sr_8_Cu_5_O*_x_* (4805). Results of the synthesis of the Pb-doped 2212 phase showed the presence of the (Ca, Sr)_2_PbO_4_ impurity. With trial amounts of Pb = 0.1, 0.2 and 0.3 in Bi_2−_*_x_* Pb*_x_*Sr_1.5_Ca_1.5_Cu_2_O*_x_*, it was found that Pb substitutes at the ≈ 0.1 mol level into the Bi site, and, subsequently, the Pb = 0.1 sample was used (Table 1). Lead was found not to substitute in the 4805 phase, and the 4805 phase was not in equilibrium with 2223. The other phases (2310, 119*x*5, and 2201) all form solid solutions with Pb at a ratio of 1.8/0.4.

##### 1.2.2.3. Five Phase Equilibrium Volumes of 2223–2212

Based on the 11 phases that are in equilibrium with the 2223 phase, the possibility of various five-phase volumes is immense. According to the combinatorial formula

C=k!/[m!(k−m)!], (1)

which gives the number of combinations *C* of *k* objects taken *m* at a time, there are 330 potential five-phase combinations that contain four of the 11 phases with 2223. This is a number too large to handle by trial and error. However, the number of possibilities for initial investigation was narrowed considerably by choosing only combinations that contain both 2212 and 2223 phases. The number of initial possibilities is thereby reduced by a factor of nearly 3. Because of the requirements for self consistency of these volumes, for example, to avoid overlapping phase space, the number of possibilities that remained for evaluation diminished rapidly as experimental data were accumulated. Coexistence with 2212 phase was chosen to be studied first, not only because it is an important 80 K superconductor, but also because during the formation of the 2223 phase, the 2212 phase is one of the precursors formed before the 2223 phase appears [34,35,43]. Furthermore, processing of the 2223 phase often results in the presence of the 2212 phase. A precise knowledge of the relationships of the 2212 and 2223 phases with other phases will significantly enhance our understanding of 2223-phase formation and processing. Sixteen such volumes were determined. After the 2223 + 2212 subsolidus was determined, the remaining 2223 subsolidus volumes could be added in a similar way.

### 1.3. General Approach to Obtaining the Primary Phase Field

By definition, a primary phase is the first crystalline phase to appear on cooling a composition from the liquid state. A primary phase field is the locus of all compositions in a phase diagram having a common primary phase. The primary phase field of a binary phase is a line, that of a ternary phase is a surface, and that of a quaternary phase can be described as a volume. Therefore, in the four-component Pb-free BSCCO system, the primary phase field of the 2212 phase is represented by a volume, and for the 2223 phase, the primary phase field is a multidimensional volume.

In order to obtain the primary phase field of a given phase of interest, the first step is to determine all compounds that are in equilibrium with the phase of interest. Next, the multiphase compatibility regions involving this phase are identified. For example, in a ternary system, one would determine all three-phase compatibility regions involving the ternary phase of interest, and four-phase compatibility regions in a quaternary system, and five-phase volumes in a five-component system, respectively. For each of the compatibility regions, the onset melting temperature is then determined. The compositions of the first liquid formed in each of these regions will determine the outline of the primary phase field. The method of locating the primary phase field has been illustrated and discussed in detail previously with reference to the Pb-free 2212 phase [26].

## 2. Experimental Method

Samples for this investigation were prepared by the solid-state calcining technique. Stoichiometric starting mixtures of PbO, Bi_2_O_3_, SrCO_3_, CaCO_3_, and CuO were homogenized, pressed, and heat treated at temperatures corresponding to the stable range of phase formation, based on available phase diagram information. Approximately 30 g of the 2223 phase were prepared by using the composition Bi_1.8_Pb_0.4_Sr_2_Ca_2.2_Cu_3_O*_z_*. The heat treatment process for this composition, which was performed in air, was as follows: 840 °C, 24 h; 850 °C, 24 h; 855 °C, 40 h; 860 °C, 40 h; and 855 °C, 120 h. Powder x-ray diffraction was used to confirm the phase formation.

### 2.1. Five-Phase Equilibria Involving the Pb-2223 Phase

The eleven phases as discussed above were used as starting materials for preparing five-phase mixtures that contained the 2223 phase. Table 2 lists the 80 five-phase samples which we have studied for determining a complete set of five-phase volumes. These samples were prepared by mixing approximately equal volumes of the phases, pelletizing, and calcining them at 810 °C–820 °C for 2 days in a 7.5 % O_2_ atmosphere, followed by grinding, repelletizing, and reheating at the same temperature for another 2 days with intermediate grindings. The choice of temperature range for calcining was based on the report by Carter et al. [67] that the thermal stability of the 2223 phase contained within a Ag sheath extended over the range of ≈ 805 °C to 835 °C at $p_{O_{2}}=7.61\times 10^{3}\text{MPa}$ (0.075 atm), and that it decomposed to 2212, CuO, and Ca_2_PbO_4_ at 800 °C [22]. After heat treatment, all samples were subjected to powder x-ray diffraction to confirm that the five-phase compatibilities persisted.

### 2.2. Determination of Liquid Composition

The procedure for obtaining compositions of melts that are produced during initial melting of multiphase volumes has been documented elsewhere [26]. The various steps can be summarized as follows: (1) DTA/thermogravimetric analysis studies were conducted to obtain an indication of thermal events. Initial melting temperatures of the five-phase assemblages were measured by differential thermal analysis (DTA) with ≈ 50 mg of annealed sample contained in a MgO crucible at a scan speed of 4 °C/min under a flowing atmosphere of 7.5 % O_2_/92.5 % Ar. Melting temperatures (during the heating cycle) obtained by DTA were chosen as the intercept of the extrapolated baseline with the linearized slope of the rising peak. (2) To capture liquid, a small piece of MgO wick was mixed in with the sample, which was placed in a MgO crucible and annealed in the appropriate atmosphere (air, or volume fraction of 7.5 % O_2_ + 92.5 % Ar). (3) Samples were annealed in purified air and were quenched in liquid-nitrogen-cooled helium for further characterization. (4) Powder x-ray characterization was performed on solid residual phases and on selected wick material to identify the crystallized melt. (5) SEM examination and x-ray mapping were conducted to study the microstructure of the quenched materials. (6) Quantitative energy dispersive x-ray spectrometry (EDS) was applied to obtain the composition of the melt captured in the quenched wick and the compositions of crystalline phases. The EDS data were reduced according to conventional methods [99] via the DTSA software package [100], which incorporates several advanced features for spectral manipulation and quantification. The standards used for microanalysis were Bi_2_Sr_1.5_Ca_1.5_Cu_2_O*_z_* and (Pb, Zr)TiO_3_. Analytical uncertainties (one standard deviation) are estimated at < 10 % relative. Uncertainties in DTA temperatures quoted in this paper are estimated a < ± 7 °C (one standard deviation).

## 3. Results and Discussion

This section focuses on the description of the complicated five-phase equilibrium volumes, the first liquids that are associated with these volumes, and the construction of the Pb-2223 primary phase field.

### 3.1. Five-Phase Compatibilities of Pb-2223 at 810 °C to 820 °C in 7.5 % O_2_

The compounds in the Bi-Pb-Sr-Ca-Cu-O system include a complicated series of binary, ternary, quaternary, and five-component solid solutions. Therefore, a large number of two phase tie-line bundles and stacks of three-phase tie planes exist. In the following discussion, the exact equilibrium compositions of the solid solutions that participate in specific equilibria are not specified. Also, because of the large number of phases, and closely spaced phase compositions, the 2223 phase compatibilities include a number of relatively “flat,” or shallow, five-phase equilibrium volumes. This implies that in certain regions of the phase diagram, a small variation in composition or temperature can lead to a dramatic change in the phase equilibrium assemblage.

Among the 80 five-phase starting mixtures that were prepared and annealed at 810 °C to 820 °C in 7.5 % volume fraction of O_2_, 29 five-phase volumes that involve the 2223 phase were found to be mutually stable in a topologically consistent manner, as indicated in Table 2. The volumes which are marked with (^a^) were those that form a consistent set. The remaining volumes were found to either result in a smaller number of components, or to be metastable as a result of reactions to produce other assemblages or because of conflict with the remaining set. There are a few volumes (marked with the symbol “M”), however, that also contain a small amount of the 2212 phase in addition to the reported five phases.

These 29 subsolidus volumes are consistent with each other in that each volume shares a given side with no more than one other (Table 3). Among them, there are a total of 16 volumes which contain of 2223-2212 as a pair. Because the 2212 and 2223 phases have similar structures (members of the same homologous series), and the 2212 phase is a precursor for the formation of the 2223 phase, it is not surprising that their mutual solid-state compatibilities are extensive. There are a total of nine five-phase volumes that contained the 2223 + CuO pair, nine five-phase volumes that contain the 2223 + 014x24 pair, and four five-phase volumes that contain the 2223 + (Ca, Sr)_2_CuO_3_ pair. The number of volumes for the latter two pairs is much smaller than for the 2223 + 2212 pair. This is so because CuO and (Ca, Sr)_2_CuO_3_ are one- and three-component oxide phases, respectively, whereas 2212 is a five-component phase (when it contains lead) that lies nearer to the center of the system. This gives it access to a much larger number of phases. The 1*x*20 ([Ca, Sr]_2_PbO_4_) phase was also found to have a wide stability region, and occurred in eight of the 16 volumes. Consequently, this phase is often found to be an impurity phase, along with the 2212 phase, during the preparation of 2223.

A comparison of the five-phase volumes of Table 3 with results in the existing literature indicates both similarities and differences. For example, Kaesche et al. [56, 80] reported two five-phase volumes involving the 2223 phase: 0*x*21-CuO-014*x*24-2212-2223 and 3221-014*x*24-CuO-2212-2223. Our results agree with the first volume; however, we found that the latter volume was converted into the 2223-2212-014*x*24-CuO-119*x*5 assemblage. Undoubtedly, many factors influenced the experimental results, including the sensitivity of the phase assemblages to processing conditions, the sluggish kinetics of phase formation, and the very closely spaced phase stability fields of the high-*T*_c_ phases. Furthermore, our samples were studied under 7.5 % volume fraction of O_2_ at ≈ 810 °C and the samples of Kaesche et al. [56, 80] were investigated in air (22.1 % O_2_). This difference in conditions may be the cause of the differences in the observed equilibria.

### 3.2. Initial Melting of the Five-Phase Volumes

The DTA temperatures (in 7.5 % O_2_) of initial melting for each of the 29 five-phase volumes are also listed in Table 3, along with the temperatures at which the sample was quenched, and the compositions of initial melts that are based on EDS analyses of the MgO wicks. These DTA temperatures range from 816 °C for the assemblage 2223-CuO-1*x* 20-0*x* 11′-014*x* 24, to 865 °C for the assemblage 2223-2212-3221-CaO-2310. This spread of approximately 40 °C is much smaller than the ≈ 70 °C spread observed in the four-phase volumes that contain the Pb-free 2212 phase [26].

The solidus of the five-phase volumes is defined by the temperatures at which the first liquids appear on heating. The solidus temperatures are useful for two main purposes. They indicate the maximum temperatures available for equilibrium solid-state processing of 2223 in each five-phase volume, and they also indicate the range of temperatures over which equilibrium liquids are available for 2223 processing. Below 816 °C, no stable equilibrium liquids are available for 2223 processing in equilibrium with 2212. Above 865 °C, which is the highest initial melting temperature among these volumes, the compositions for which 2223 and liquid are in equilibrium with each other are severely restricted due to the expansion of the liquid field.

The range of melt compositions for these volumes expressed in mole fractions is BiO_1.5_, 7.3 % to 28.0 %; PbO, 1.2 % to 19.4 %; SrO, 11.3 % to 27.8 %; CaO, 9.8 % to 30.8 %; and CuO, 17.1 % to 47.0 %. Although PbO is present in the liquid of every volume, the concentration of PbO is the least among the five oxide components, followed by SrO. The concentration of the CuO component has the highest value. The subsolidus volumes with relatively high PbO concentration give rise to higher PbO content in the melt. For example, in the five-phase volume 2223-0*x*11′-3221-1*x*20-0*x*11, which contains the PbO-rich phases Bi_0.5_Pb_3_Sr_2_Ca_2_CuO_z_ and (Ca_1.9_Sr_0.1_)PbO_4_, the PbO concentration is relatively high (18.7 %). By contrast, for the volume 2223-2212-0*x*11-0*x*11′-CuO, where there is no PbO-containing compound other than a small amount of Pb substituted in the 2223, 2212 and 119x5 phases, the PbO content of the liquid is only 1.2 %.

### 3.3. The Primary Phase Field of the 2223 Phase

By analogy with our determination of the primary phase volume of the Pb-free phase [26], the 29 compositions of the five-phase volumes were modeled using a computational geometry technique based on forming a convex hull from the experimentally determined chemical compositions. This mathematical notion of a convex hull, the smallest convex set of points that contains all of the given data points, has been used in many physical science applications. In this case, the convex hull represents the extent of the compositional volume. This numerical procedure results in a well-formed, hyper-volume in five-dimensional space. It is important to note the requirement that chemical compositions sum to unity results in the loss of one degree of freedom. This explains the flat appearance of the three dimensional cross-sections. The convex hull is defined by the matrix equation

Ax+b≤0, (2)

where ***A*** is a matrix whose rows define the unit normal vectors to the faces of the convex hull. Each element of the vector ***b*** defines the proximity of the given face to the origin (in this five-dimensional space). The vector ***x*** gives the coordinates corresponding to a given point. The matrix ***A*** and vector ***b*** can be found using a so-called “sweeping algorithm” that has been implemented and tested [101]. When the procedure is complete, the matrix ***A*** has dimension *k* × 5 and the vector ***b*** has dimension ***k*** where ***k*** is the number of faces in the convex hull.

### 3.4 Graphical Representations

#### 3.4.1. Subsolidus Phase Relationships

**Fig. 1:**
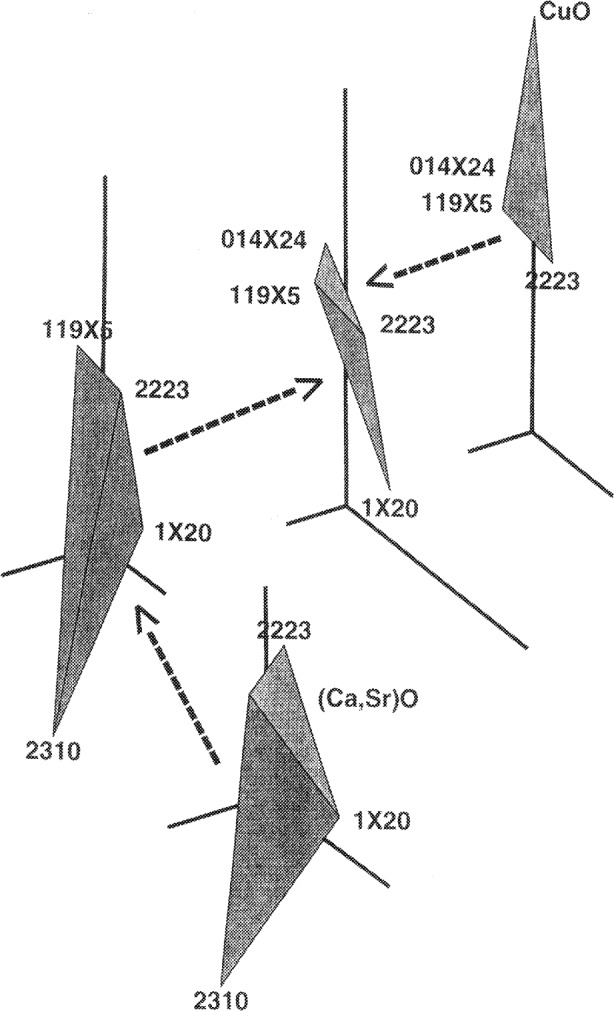
An example of four of the 16 five-phase subsolidus assemblages, as projected through the 2212 phase. Arrows indicate how volumes are interconnected in these exploded views.

Since all of the 29 volumes in Table 3 are expressed in the five-component space, representing them is not straightforward. One way to view them is via a thermodynamic method, which involves projecting through the composition of a phase common to a group of assemblages. However, not all 29 volumes can be viewed simultaneously in this manner. In other words, one can view groups of volumes which contain a common compound plus the 2223 phase individually. As an example, the sixteen 2223-2212 volumes can be viewed together as a group [87]. For convenience, one can recast the coordinate system of simple conventional oxides into a system using 2310 (i.e., Bi_2_Sr_3_CaO_7_), PbO, 2212 (e.g., Bi_2.0_Pb_0.2_Sr_2_CaCu_2_O*_z_*), CaO, and CuO as reference according to the following equations:

$$\begin{array}{l} n_{2212}=1.5\left(n_{\text{BiO}1.5}\right)-1.0\left(n_{\text{SrO}}\right)\\ n_{\text{Pb}O^{\prime }}=-0.3\left(n_{\text{BiO}1.5}\right)+1.0\left(n_{\text{PbO}}\right)+0.2\left(n_{\text{SrO}}\right)\\ n_{2310}=-1.0\left(n_{\text{BiO}1.5}\right)+1.0\left(n_{\text{SrO}}\right)\\ n_{\text{Ca}O^{\prime }}=-0.5\left(n_{\text{BiO}1.5}\right)+1.0\left(n_{\text{CaO}}\right)\\ n_{\text{Cu}O^{\prime }}=-0.3\left(n_{\text{BiO}1.5}\right)+2.0\left(n_{\text{SrO}}\right)+1.0\left(n_{\text{CuO}}\right), \end{array}$$ (3)

where the quantity *n* is the amount of substance for each indicated component (The SI unit for *n* is mole). Projection through 2212 into a three-dimensional space is then carried out. The resulting sixteen volumes were found to fit well with each other in the 3-dimensional space without overlapping. Figure 1 shows how four out of the sixteen volumes fit with each other.

#### 3.4.2. Primary Phase Field

**Fig. 2:**
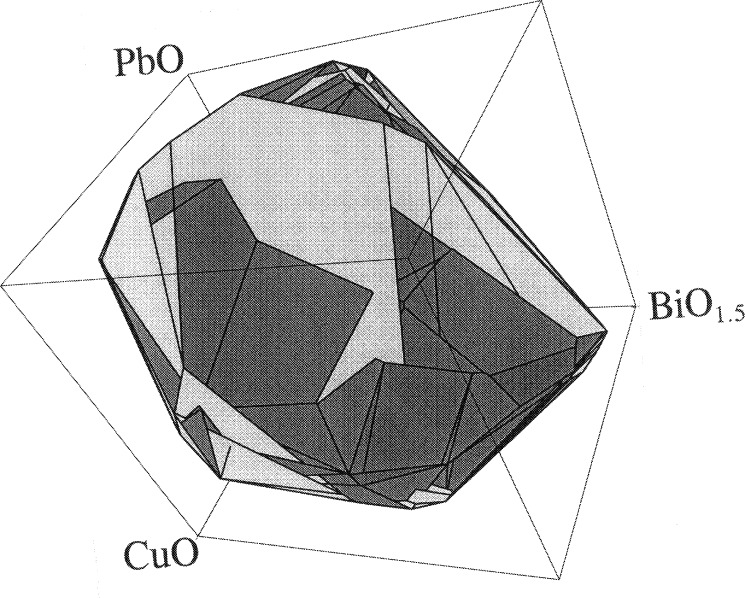
The primary crystallization field of the Pb-2223 phase. An isoplethal section made by holding the BiO_1.5_ and PbO values constant at the median mole fraction values for the 29 data points (BiO_1.,5_ = 20.4 %, and PbO = 6.9 %).

To graphically represent the primary phase field and give a general sense of its “shape,” we employ an isopleth projection technique. In this technique the five-dimensional convex hull is viewed in three-dimensions by holding two components of the composition fixed at their mean value. Figure 2 shows an isoplethal section made by holding the BiO_1.5_ and PbO values constant at the median values for the 29 data points, with the remaining oxides shown on the three Cartesian axes. The view of the section of the 2223 primary phase field in Fig. 2 appears to have a broad oval shape.

It is interesting that the mean of the compositional analyses in Table 3 is close to the 2223 composition. Obviously, since 2223 melts incongruently, the 2223 composition cannot plot within the primary phase field. The position of the mean suggests that there may be more structure to the primary phase field than has been accounted for by the convex hull. There are two possibilities. If the primary phase field has a strongly curved concavo-convex shape about the 2223 stoichiometry, without actually enclosing it, then the convex hull fitting procedure, which doesn’t allow for concavity, would fit a single surface around the entire ensemble. Alternatively, if the primary phase field actually consisted of a two-liquid field, split on opposite sides of the 2223 stoichiometry, the convex hull would again fit a single surface. These possiblities are undergoing further evaluation in our laboratories.

### 3.5. Application of the Primary Phase Field

The primary phase field provides a compositional region that one can use for crystal growth and for melt processing. In principle, when a composition is prepared within the limits defined by the primary phase field, crystals of 2223 will be the first to crystallize from the melt.

**Fig. 3:**
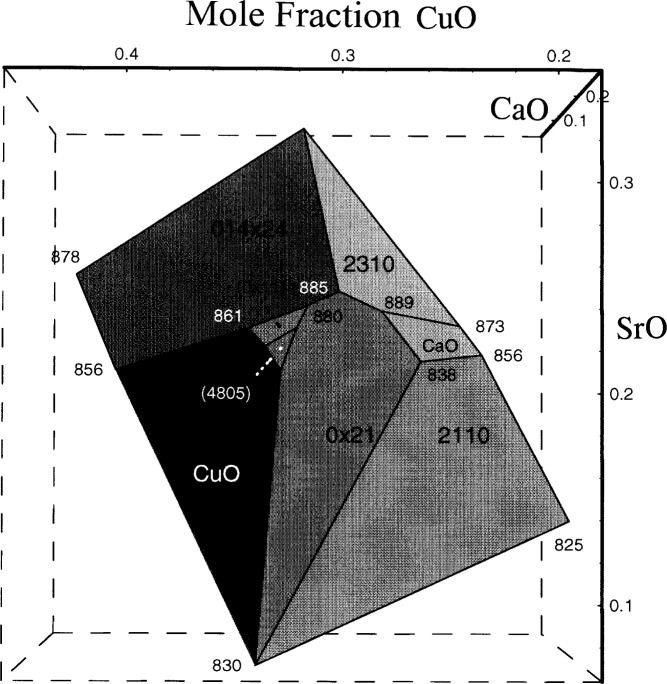
Primary phase field of the 2212 phase in the BSCCO system. The onset melting temperatures are indicated at the corners.

We have demonstrated previously that in the process of growing the Pb-free 2212 crystals [102], when one starts with a composition prepared within the primary phase field as shown in Fig. 3, even with a crystallization scheme using relatively rapid cooling rate (0.5 °C/min), we were able to obtain a sample with microstructure mostly dominated by the 2212 crystals. On the other hand, a composition that was prepared outside this field gave a microstructure that was mostly the calcium cuprate phase, with only a very small amount of the 2212 phase present. Applying the similar principle during the processing of the 2223 phase, compositions falling outside the primary phase field should be avoided, as they would be expected to produce large primary crystals of unwanted phases.

Equation (2) can be used to determine if a given composition lies inside the Pb-2223 primary phase field. If the difference of the two terms on the left side of the equation (***Ax* − *b*** ≤ 0) is negative, the particular composition lies inside the crystallization volume. If the value of all components is zero, the composition is on the surface; and if the value of any one component is positive, it is outside the volume. Compositions falling outside this volume should be avoided for the processing of the 2223 phase, as they would be expected to produce large primary crystals of unwanted phases. Table 4 shows an example of two compositions in order to illustrate whether the composition lies relative to the primary phase field. The first composition was found to lie inside the volume because from the matrix manipulation, all components of ***Ax* − *b*** were found to be negative. The second composition was found to be outside the volume because 68 out of 200 elements are positive.

## 4. Summary and Future Needs

We have illustrated the general procedure of obtaining the primary phase fields of the five-component Pb-2223 phases. The subsolidus phase equilibria and liquid melt compositions of a complete set of 29 five-phase volumes that contain the Pb-2223 phase were obtained. These volumes are consistent with each other. The liquid compositions of the initial melts of these five-phase volumes have been measured and were used to construct the primary phase field. This field represents compositional regions where liquids can be found in equilibrium with the superconductors. It was found that, for these 29 volumes, the range of initial melt compositions expressed in mole fractions encompasses BiO_1.5_: 7.3 % to 28.0 %; PbO: 1.2 % to 19.4 %; SrO: 11.3 % to 27.8 %; CaO: 9.8 % to 30.8 %; and CuO: 17.1 % to 47.0 %. A PbO component is present in the initial melt of every volume. The subsolidus volumes that contain PbO-rich phases give rise to melts with high PbO content.

The World Wide Web provides an excellent opportunity to give access to the primary phase field model described here. Presently, work is underway to construct a website where users can input various chemical compositions corresponding to experiments and, using java-based web technology, the convex hull procedure described here can be implemented and results displayed immediately. In this way, a user (potentially anywhere in the world) can employ this technique. The resulting polyhedra can be revolved in order to be viewed from different angles and used to make decisions relating to the shape. Also, it can be determined whether a trial composition is within the polyhedron.

Silver has been reported to enter the liquid phase, but, there was no indication that Ag enters into the 2223 phase under subsolidus subsolidus conditions [18, 98].

## Figures and Tables

**Table 1:** Symbols and compositions of compounds prepared and used in this study

Symbol	([Bi,Pb]:Sr:Ca:Cu)	Representative compositions
2223	(2:2:2:3)	(Bi_1.8_Pb_0.4_)Sr_2_Ca_2.2_Cu_3_O_z_
2212	(2:2:1:2)	(Bi_1.9_Pb_0.1_)Sr_1.5_Ca_1.5_Cu_2_O_z_
014*x*24	(0:14:*x*:24)	Sr_7_Ca_7_Cu_24_O_41_
0*x*21	(0:*x*:2:1)	(Ca_1.9_Sr_0.1_)CuO_3_
3221	(3:2:2:1)	Bi_0.5_Pb_3_Sr_2_Ca_2_CuO_z_
2310	(2:3:1:0)	Bi27.82Pb_6.18_Sr_49.5_Ca_16.5_O_z_
2201	(2:2:0:1)	Bi_1.64_Pb_0.36_Sr_2_CuO_z_
119*x*5	(11:9:*x*:5)	Bi_1.8_Pb_0.4_Sr_1.6_Ca_0.2_CuO_z_
0*x*11	(0:*x*:1:1)	(Ca_0.86_Sr_0.14_)CuO_2_
0*x*11′	(0:*x*:1:1′)	(Ca_0.5_Sr_0.5_)CuO_2_
4805	(4:8:0:5)	Bi_3.4_Pb_0.72_Sr_8_Cu_5_O_z_
CaO		CaO
CuO		CuO
1*x*20	(l:*x*:2:0)	(Ca_1.9_Sr_0.1_)PbO_4_

**Table 2:** Results of heat-treatment of 81 five-phase mixtures

Starting composition	X-ray analysis of results
2223-2212-1*x*20-119*x*5-2310^a^	2223-2212-1*x*20-119*x*5-2310
2223-2212-1*x*20-2310-CaO^a^	2223-2212-1*x*20-2310-CaO
2223-2212-0*x*21-1*x*20-CuO^a^	2223-2212-0*x*21-1*x*20-CuO
2223-2212-0*x*21-3221-CuO	2223-2212-0*x*21-1*x*20-CuO
2223-2212-1*x*20-CuO-CaO	2223-2212-0*x*21-1*x*20-CuO
2223-2212-1*x*20-0*x*11-3221^a^	2223-2212-1*x*20-0*x*11-3221
2223-2212-1*x*20-CuO-*x*11^a^	2223-2212-1*x*20-CuO-0*x*11
2223-2212-0*x*21-014*x*24-CuO^a^	2223-2212-0*x*21-014*x*24-CuO
2223-2212-014*x*24-1*x*20-119*x*5^a^	2223-2212-014*x*24-1*x*20-119*x*5
2223-2212-11*x*5-1*x*20-0*x*11	2223-2212-1*x*20-119*x*5-014*x*24
2223-2212-2310-0*x*11-3221^a^	2223-2212-2310-0*x*11-3221
2223-2212-119*x*5-0*x*11′-CuO^a^	2223-2212-119*x*5-0*x*11-CuO
2223-2212-014*x*24-119*x*5-CuO^a^	2223-2212-014*x*24-119*x*5-CuO
2223-2212-014*x*24-3221-CuO	2223-2212-014*x*24-CuO-119*x*5
2223-2212-0*x*11-014*x*24-CuO	2223-2212-014*x*24-CuO-119*x*5
2223-2212-014*x*24-0*x*21-1*x*20^a^	2223-2212-014*x*24-0*x*21-1*x*20
2223-2212-0*x*21-1*x*20-014*x*24	2223-2212-0*x*21-1*x*20-014*x*24
2223-2212-3221-CaO-1*x*20^a^	2223-2212-3221-CaO-1*x*20
2223-2212-CaO-0*x*11′-3221	2223-2212-3221-CaO-1*x*20
2223-2212-0*x*11-2310-119*x*5^a^	2223-2212-0*x*11-2310-119*x*5
2223-2212-3221-CaO-2310^a^	2223-2212-3221-CaO-2310
2223-2212-3221-0*x*11′-119*x*5	2223-2212-3221-CaO-2310
2223-2212-0*x*11-0*x*11′-2310^a^	2223-2212-0*x*11-0*x*11′-2310
2223-2212-3221-0*x*11′-0x11	2223-2212-0*x*11-0*x*11′-2310
2223-2212-0*x*11-0x11′-CuO^a^	2223-2212-0*x*11-0*x*11′-CuO
2223-1*x*20-CuO-0*x*21-014*x*24^a^	2223-1*x*20-CuO-0*x*21-014*x*24
2223-1*x*20-CaO-0*x*11′-2310^a^	2223-1*x*20-CaO-0*x*11′-2310
2223-1*x*20-CaO-0*x*11′-3221^a^	2223-1*x*20-CaO-0*x*11′-3221
2223-CuO-1*x*20-0*x*11′-014*x*24^a^	2223-CuO-1*x*20-0*x*11′-014*x*24
2223-2310-1*x*20-014*x*24-0*x*11′^a^	2223-2310-1*x*20-014*x*24-0*x*11′
2223-0*x*11′CuO-1*x*20-0*x*11^a^	2223-0*x*11′CuO-1*x*20-0*x*11
2223-0*x*11′-3221-1*x*20-0*x*11^a^	2223-0*x*11′-3221-1*x*20-0*x*11
2223-0*x*11′-CaO-0*x*11-3221^a^	2223-0*x*11′-CaO-0*x*11-3221
2223-0*x*11′-CaO-2310-0*x*11^a^	2223-0*x*11′-CaO-2310-0*x*11
2223-0x11′-2310-0*x*11-119*x*5^a^	2223-0*x*11′-2310-0*x*11-119*x*5
2223-0*x*11′-2310-119*x*5-014*x*24^a^	2223-0*x*11′-2310-119x5-014*x*24
2223-0*x*11′-014*x*24-119*x*5-CuO^a^	2223-0*x*11′-014*x*24-119*x*5-CuO
2223-2310-3221-CaO-0*x*11^a^	2223-2310-3221-CaO-0*x*11
2223-1*x*20-CuO-014*x*24-119*x*5	2223-2212-1*x*20-CuO
2223-1*x*20-CuO-0*x*11-119*x*5	2223-2212-1*x*20-CuO
2223-1*x*20-0*x*11-119*x*5-2310	2223-2212-1*x*20-2310
2223-1x20-0x11-2310-3221	2223-2212-1*x*20-0*x*11
2223-1*x*20-CaO-3221-2310^a^	2223-2212-1*x*20-CaO-2310
2223-CaO-3221-2310-1*x*20	2223-2212-1*x*20-2310-CaO
2223-1*x*20-119*x*5-014*x*24-2310	2223-2212-2310-1*x*20-119*x*5
2223-1*x*20-CuO-014*x*24-2310	2223-2212-1*x*20-CuO
2223-1*x*20-CuO-0*x*11-2310	2223-2212-1*x*20-CuO
2223-CuO-0*x*11-119*x*5-014*x*24	2223-2212-2310-119*x*5
2223-CaO-2310-3221-0*x*11	2212-3221-CaO-2310
2223-2310-0*x*11-014*x*24-119*x*5	2223-2212-0*x*11-2310-119*x*5
2223-2310-1*x*20-CuO-0*x*11	2223-2212-1*x*20-CuO-0*x*11
2223-2310-0*x*11-CuO-119*x*5	2223-2212-CuO-119x5
2223-2310-1*x*20-CuO-014*x*24	2223-1*x*20-CuO-014*x*24
2223-2310-0*x*11-CuO-014*x*24^b^	2223-CuO-2310-0*x*11
2223-2212-0*x*11-0*x*11′-119*x*5	2223-2212-0*x*11-0*x*11′
2223-119*x*5-2310-1*x*20-0*x*11′	2223-2212-1*x*20-2310
2223-014*x*24-1*x*20-0*x*11′-CuO	2223-2212-1*x*20-CuO-0*x*21
2223-2212-1*x*20-0*x*21-3221	2223-2212-1*x*20-3221
2223-2212-0*x*11′-0*x*11-119*x*5	2223-2212-0*x*11′-0*x*11
2223-2212-0*x*11-CuO-119*x*5	2223-2212-0*x*11-CuO
2223-2212-0*x*11-0*x*21-3221	2223-2212-3221-0*x*11
2223-2212-3221-0*x*11-CuO	2212-2310-0*x*11-119*x*5
2223-2212-1*x*20-0*x*11′-CuO	2223-2212-1*x*20-CuO
2223-2212-2310-CuO-1*x*20	2223-2212-1*x*20-CuO
2223-2212-1*x*20-119*x*5-CaO	2212-CaO-2310-1*x*20
2223-2212-1*x*20-0*x*11-119*x*5	2223-2212-1*x*20-0*x*11
2223-2212-014*x*24-3221-1*x*20	2223-2212-014*x*24-1*x*20
2223-2212-1*x*20-3221-CuO	3221-CuO-119*x*5
2223-2212-014*x*24-3221-CuO	3221-CuO-119*x*5-014*x*24
2223-2212-014*x*24-0*x*21-3221	2212-3221-014*x*24-0*x*21
2223-2212-1*x*20-119*x*5-3221	2212-3221-1*x*20-119*x*5
2223-2212-119*x*5-3221-CuO	2212-119*x*5-3221-CuO
2223-2212-3221-0*x*11-119*x*5	2223-2212-3221-0*x*21
2223-2212-014*x*24-1*x*20-CaOb	2223-2212-014*x*24-1*x*20-CaO
2223-2212-1*x*20-CaO-0*x*11b	2223-2212-1*x*20-CaO-0*x*11
2223-2212-014*x*24-0*x*11-1*x*20	2223-2212-0*x*11-1*x*20
2223-2212-CaO-2310-0*x*11′	2223-2212-0*x*11′-2310
2223-2212-0*x*11′-119*x*5-2310	2223-2212-2310-CaO
2223-2212-2310-3221-014*x*24	2223-2310-3221-014*x*24
2223-2212-2310-CaO-0*x*11′	2223-2212-2310-CaO

a Samples interpreted as representing equilibrium subsolidus volumes.

b Metastable volumes.

**Table 3:** Twenty-nine five-phase equilibrium volumes that contain the 2223 phase prepared in 7.5 % volume fraction O_2_. Differential thermal analysis (DTA) temperatures indicate initial melting, quench temperatures (Qch) indicate temperature of the melt wick sampling experiments

Five-phase equilibrium^a^		DTA *t* (°C)^b^	Qch *t* (°C)^c^	Melt composition (mole fraction, %)^d^				
				BiO_1.5_	PbO	SrO	CaO	CuO
1	2223-2212-1*x*20-119*x*5-2310	827	830	23.6	7.6	26.3	25.4	17.1
2	2223-2212-1*x*20-2310-CaO	830	835	23.0	7.3	27.1	23.5	19.1
3	2223-2212-0*x*21-1*x*20-CuO	837	840	18.5	14.4	16.3	14.2	36.6
4	2223-2212-1*x*20-0*x*11-3221	838	842	19.9	14.0	19.9	15.4	30.8
5	2223-2212-1*x*20-CuO-0*x*11	839	842	15.0	10.0	16.0	12.0	47.0
6	2223-2212-0*x*21-014*x*24-CuO	845	850	28.0	3.2	17.1	21.6	30.1
7	2223-2212-014*x*24-1*x*20-119*x*5	840	845	20.2	6.5	20.8	22.2	30.3
8	2223-2212-2310-0*x*11-3221	842	845	13.5	4.1	27.8	26.4	28.2
9	2223-2212-119x5-0*x*11-CuO	842	846	17.2	2.7	19.9	28.7	31.5
10	2223-2212-014*x*24-119*x*5-CuO	845	850	21.4	2.8	24.0	20.9	30.9
11	2223-2212-014*x*24-0*x*21-1*x*20	846	851	17.0	11.0	16.2	17.9	37.9
12	2223-2212-3221-CaO-1*x*20	848	852	19.3	10.2	23.7	18.4	28.4
13	2223-2212-0*x*11-2310-119*x*5	850	853	21.6	4.7	24.5	26.2	23.0
14	2223-2212-0*x*11-0*x*11′-CuO	850	855	21.8	1.2	21.7	18.3	37.0
15	2223-2212-0*x*11-0*x*11′-2310	853	858	25.3	2.3	22.7	17.3	32.4
16	2223-2212-3221-CaO-2310	865	870	20.4	7.6	20.9	25.8	25.3
17	2223-1*x*20-CuO-0*x*21-014*x*24	828	831	10.4	18.2	11.3	20.6	39.5
18	2223-1*x*20-CaO-0*x*11′-2310	832	835	20.8	12.8	23.5	15.4	27.5
19	2223-1*x*20-CaO-0*x*11′-3221	831	833	15.8	16.9	24.1	15.2	28.0
20	2223-CuO-1*x*20-0*x*11′-01424	816	818	13.8	16.9	20.3	15.7	33.4
21	2223-2310-1*x*20-014*x*24-0*x*11′	832	835	18.8	15.9	23.4	12.0	29.9
22	2223-0*x*11′CuO-1*x*20-0*x*11	838	841	7.3	16.7	12.6	20.4	43.0
23	2223-0*x*11′-3221-1*x*20-0*x*11	820	823	16.6	18.7	18.3	14.1	32.3
24	2223-0*x*11′-CaO-0*x*11-3221	830	833	17.0	19.4	15.1	15.3	33.2
25	2223-0*x*11′-CaO-2310-0*x*11	840	845	12.4	1.6	26.5	30.8	28.7
26	2223-0*x*11′-2310-0*x*11-119*x*5	848	853	27.6	2.1	24.8	13.4	32.1
27	2223-0*x*11′-2310-119*x*5-014*x*24	827	823	19.9	4.1	23.0	15.8	37.2
28	2223-0*x*11′-014*x*24-119*x*5-CuO	840	845	23.4	1.6	24.1	10.5	40.4
29	2223-2310-3221-CaO-0*x*11	838	840	15.4	16.9	25.2	9.8	32.7

a See Table 1 for symbols and compositions.

b Indicates initial melting; combined standard uncertainty < 6 °C.

c Indicates temperature of melt wick sampling experiments, combined standard uncertainty < 6 °C.

d For mole fractions > 10 %, the relative standard uncertainty is < 2.5 %; for mole fraction < 10 %, it is < 5.0 %.

**Table 4:** Compositions (expressed in mole fraction %) used to illustrate the use of the primary phase field of Pb-2223

Bi	Pb	Sr	Ca	Cu	*Ax* − *b*	Comments
21.82	1.20	21.66	18.32	37.01	all “−”	Inside volume
29.00	10.00	11.00	15.00	35.00	68 “+”	Outside volume
